# Impact of pulmonary rehabilitation on exercise capacity, health-related quality of life, and cardiopulmonary function in lung surgery patients: a retrospective propensity score-matched analysis

**DOI:** 10.3389/fmed.2024.1450711

**Published:** 2024-08-12

**Authors:** Chunlai Niu, Huan Lin, Zinan Zhang, Qing Wang, Yingjun Wei

**Affiliations:** ^1^Department of Respiratory and Critical Care Rehabilitation, Shanghai Second Rehabilitation Hospital, Shanghai, China; ^2^Department of Respiratory and Critical Care Medicine, The First Affiliated Hospital of Naval Medical University, Shanghai, China; ^3^Department of Thoracic Surgery, Shanghai Chest Hospital, School of Medicine, Shanghai Jiao Tong University, Shanghai, China

**Keywords:** pulmonary rehabilitation, lung surgery, exercise capacity, health-related quality of life, cardiopulmonary function

## Abstract

**Background:**

Pulmonary rehabilitation is considered beneficial for patients undergoing lung surgery, yet its specific impacts on exercise capacity, health-related quality of life (HRQL), and cardiopulmonary function require further elucidation. This study aimed to evaluate the effect of PR on these outcomes in patients undergoing lung surgery using a retrospective propensity score-matched analysis.

**Methods:**

We retrospectively analyzed 420 patients with non-small cell lung cancer (NSCLC) who underwent lung surgery from January 2022 to May 2024. Among these, 84 patients received PR while 336 did not (control group). Propensity score matching (PSM) at a 1:1 ratio yielded 46 patients in each group. Baseline characteristics, spirometry, cardiopulmonary exercise testing, respiratory muscle strength, HRQL, and muscle measurements were assessed pre-and post-surgery.

**Results:**

Before PSM, significant differences existed between groups, with the PR group being older and having different pulmonary function baselines. After PSM, groups were well-balanced. Postoperatively, the PR group showed significant improvements in FEV1/FVC (64.17% vs. 50.87%, *p* < 0.001), FEV1 (2.31 L/min vs. 1.75 L/min, *p* < 0.001), and predicted FVC percentage (88.75% vs. 68.30%, *p* < 0.001). Cardiovascular responses showed a lower CI during exercise in the PR group post-PSM (6.24 L/min/m^2^ vs. 7.87 L/min/m^2^, *p* < 0.001). In terms of exercise capacity, the PR group had higher maximal WR percentage (104.76% vs. 90.00%, *p* = 0.017) and peak VO2 (1150.70 mL/min vs. 1004.74 mL/min, *p* = 0.009). PR also resulted in less leg soreness and lower total CAT scores postoperatively. Muscle measurements indicated significantly smaller reductions in ΔHU_ESMCSA_ and percentage change in the PR group.

**Conclusion:**

Pulmonary rehabilitation significantly enhances exercise capacity, HRQL, and cardiopulmonary function in patients undergoing lung surgery. It also mitigates postoperative muscle loss, underscoring its importance in the postoperative management of lung surgery patients.

## Introduction

Lung cancer remains one of the leading causes of cancer-related morbidity and mortality worldwide, with non-small cell lung cancer (NSCLC) accounting for approximately 85% of cases (1). Surgical resection is a primary treatment modality for early-stage NSCLC, but it is often associated with significant postoperative complications and a decline in pulmonary function and exercise capacity (2). Pulmonary rehabilitation has emerged as an effective intervention to mitigate these adverse outcomes by improving respiratory muscle strength, exercise capacity, and overall quality of life in patients with chronic lung diseases (3, 4).

PR programs, which typically include exercise training, education, and psychological support, have been shown to be beneficial in various chronic respiratory conditions, including chronic obstructive pulmonary disease (COPD) and interstitial lung disease (ILD) (5, 6). These programs aim to enhance the functional status and reduce the symptom burden of patients through a multidisciplinary approach (7). In the context of lung cancer, PR is increasingly recognized for its potential to improve preoperative and postoperative outcomes, thereby enhancing recovery and reducing healthcare utilization (8, 9).

Despite the recognized benefits of PR in chronic lung diseases, its role in the perioperative management of lung cancer patients undergoing surgical resection is less well-defined. Studies have suggested that PR can improve preoperative pulmonary function and reduce postoperative complications, but comprehensive data, particularly from propensity score-matched analyses, are limited (2, 10). Given the heterogeneity in patient populations and PR program designs, there is a need for robust evidence to guide clinical practice in this setting (11).

This retrospective propensity score-matched analysis aims to evaluate the effects of PR on exercise capacity, health-related quality of life (HRQL), and cardiopulmonary function in patients undergoing lung cancer surgery. By comparing outcomes between patients who received PR and those who did not, this study seeks to provide a clearer understanding of the clinical benefits of PR in this patient population.

## Materials and methods

### Study design and patient selection

This study was a retrospective analysis of patients diagnosed with non-small cell lung cancer (NSCLC) who underwent lung cancer surgery from January 2022 to May 2024. A total of 632 patients were initially identified from the medical records of Shanghai Chest Hospital. Patients were eligible if they had histologically confirmed NSCLC, underwent VATS/minimally invasive surgery, were aged 18 years or older. Exclusion criteria included patients with missing data in their medical records, severe orthopedic or neurological impairments that precluded participation in exercise testing or pulmonary rehabilitation, significant changes in tumor size or evidence of metastasis indicating disease progression, and those who experienced severe postoperative complications. Patients with severe postoperative complications were excluded from the study to maintain a homogenous study population and to avoid confounding factors that could skew the results. Patients under the age of 18 were also excluded.

**Figure 1:**
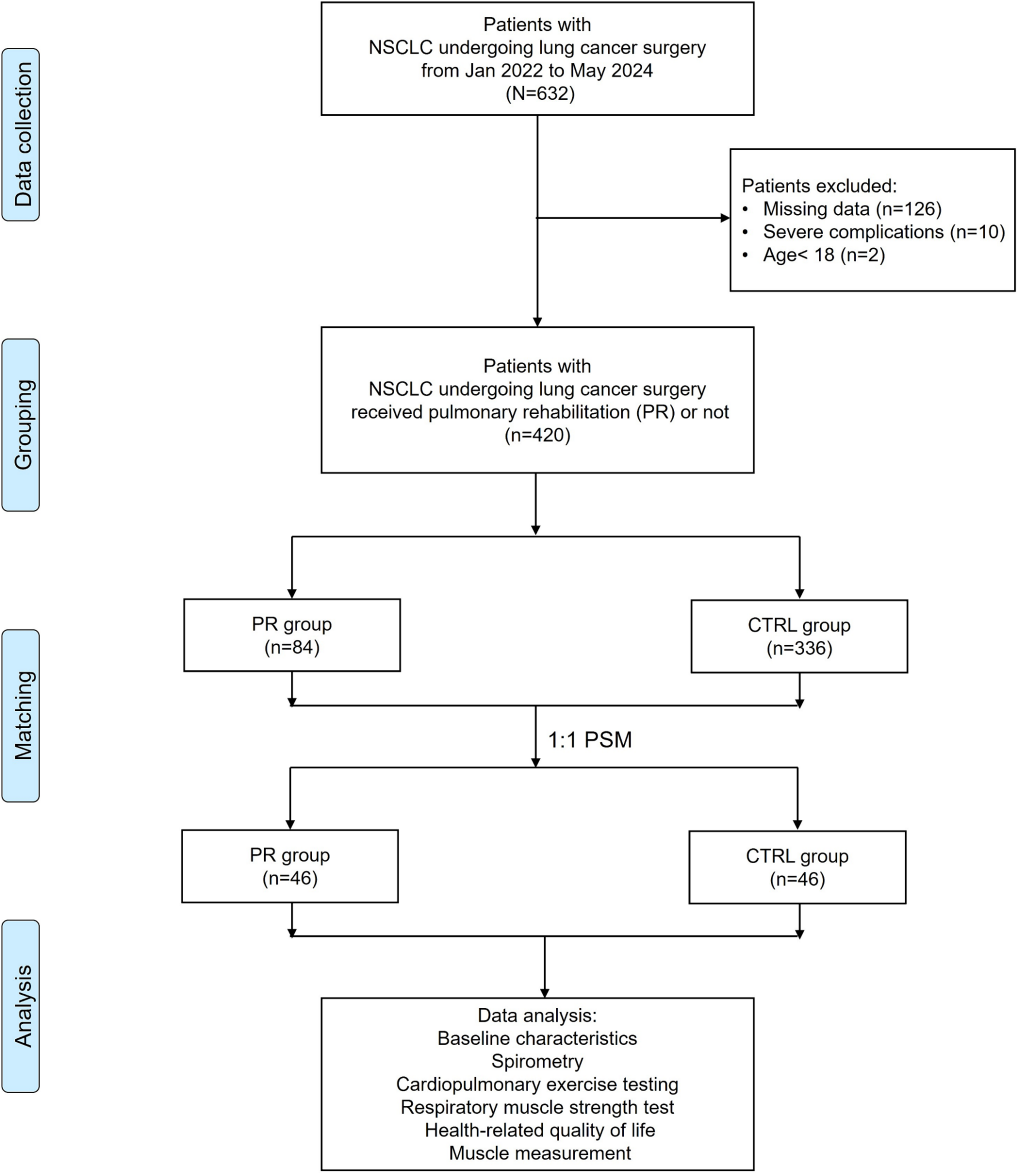
Flowchart of patient selection and grouping.

After excluding 126 patients with missing data, 10 patients with severe complications, and 2 patients under the age of 18, a total of 420 patients were eligible for further analysis. Among these, 84 patients received pulmonary rehabilitation (PR), while 336 did not (control group, CTRL). To minimize selection bias and balance baseline characteristics between the groups, propensity score matching (PSM) was employed. Patients were matched at a 1:1 ratio, resulting in 46 patients in each group. This matching process ensured comparable baseline demographics and clinical characteristics between the PR and control groups. Data analysis included baseline characteristics, spirometry, cardiopulmonary exercise testing, respiratory muscle strength, health-related quality of life (HRQL), and muscle measurements. The flowchart in Figure 1 illustrates the patient selection and grouping process.

### Ethical statement

This study was conducted in accordance with the ethical standards laid down in the Declaration of Helsinki and its subsequent amendments. The study protocol was reviewed and approved by the Ethics Committee of Shanghai Second Rehabilitation Hospital. Given the retrospective nature of the study, the requirement for informed consent was waived. However, confidentiality and privacy of the patient data were strictly maintained throughout the study. All data were anonymized prior to analysis to ensure patient confidentiality. The study aimed to provide insights that could improve clinical practice and patient outcomes, adhering to ethical principles of beneficence and non-maleficence.

### Pulmonary rehabilitation program

Pulmonary rehabilitation included a combination of exercise and education programs (2, 12), aligned with the Enhanced Recovery After Surgery (ERAS) protocol. The exercise sessions were held one to three times weekly, lasting 30–40 min each. Educational sessions were repeatedly conducted in the outpatient clinic and the exercise therapy room. The exercise regimen comprised aerobic activities (such as walking, bicycle ergometer, treadmill, and arm ergometer), strength training (focused on upper-limb exercises), flexibility exercises, and inspiratory muscle training. Educational components included guidance on smoking cessation, breathing techniques (pursed-lip, diaphragmatic, and segmental breathing), and secretion removal methods (coughing exercises, huffing, assisted coughing, and postural drainage). Exercise intensity for patients was tailored based on metabolic equivalent, peak oxygen consumption, and heart rate.

Patients participated in pulmonary rehabilitation at least once or twice before surgery. Postoperatively, their condition was reassessed 2–3 weeks after the operation, and rehabilitation was resumed. To support ongoing home-based rehabilitation, patients were provided with educational materials such as pamphlets, notes, and posters.

### Postoperative reassessment

Postoperative reassessment of the patients was conducted 2–3 weeks after surgery to evaluate their recovery and readiness to resume pulmonary rehabilitation. During this reassessment period, all mentioned tests and measurements were performed, including spirometry, cardiopulmonary exercise testing, and respiratory muscle strength measurements. These comprehensive assessments were crucial in determining the patient’s postoperative status and tailoring the subsequent rehabilitation program to their specific needs. The spirometry tests measured forced expiratory volume in 1 s (FEV1) and forced vital capacity (FVC), while the cardiopulmonary exercise tests evaluated parameters such as peak oxygen uptake (VO2), work rate (WR), and other cardiovascular responses. Respiratory muscle strength was assessed using maximum inspiratory pressure (MIP) and maximum expiratory pressure (MEP).

### Pulmonary function testing

Pulmonary function was assessed using spirometry (Medical Graphics Corp., St. Paul, MN, United States) following the guidelines of the American Thoracic Society (ATS). Parameters measured included FEV1, FVC, and the FEV1/FVC ratio.

### Cardiopulmonary exercise testing

CPET was performed using a bicycle ergometer (Lode Corival, Groningen, Netherlands) with an incremental protocol. Key variables measured included VO2, carbon dioxide output (VCO2), tidal volume (VT), and RF. HR, BP, and SpO2 were monitored simultaneously. Anaerobic threshold (AT) was determined using the V-slope method, and work efficiency (WE) was calculated by linear regression analysis of the VO2 to WR ratio. Oxygen pulse (O2P) was determined by dividing VO2 by HR, and the ventilatory equivalent (VEQ) was calculated as the ratio of VCO2 to minute ventilation (VE) at nadir during CPET.

### Respiratory muscle strength testing

MIP and MEP were measured using a respiratory pressure meter (Micro Medical Corp., England). MIP was measured after the patient exhaled to residual volume, followed by a rapid and forceful maximal inspiration. MEP was measured after the patient inhaled to total lung capacity, followed by maximal effort exhalation.

### Cardiac performance assessment

Cardiac performance, including stroke volume index (SVI) and cardiac index (CI), was measured using Physioflow (Manatec Biomedical, Poissy, France), a non-invasive hemodynamic monitoring device that uses thoracic impedance cardiography. Electrodes placed on the thorax assessed changes in impedance caused by pulsatile blood flow.

### Health-related quality of life assessment

HRQL was assessed using the Chronic Obstructive Pulmonary Disease Assessment Test (CAT), which comprises eight items evaluating symptoms such as cough, phlegm, chest tightness, breathlessness, limited activities, confidence in leaving home, sleeplessness, and energy levels. Each item is scored from 0 to 5, with higher scores indicating more severe symptoms.

### Muscle measurement

As previously described, we focused on and measured three muscles: the pectoralis, thoracic erector spinae, and lumbar erector spinae (12). We selected the erector spinae muscles at the first lumbar level for three reasons: prior studies have analyzed COPD patients using CT-based measurements of the pectoralis and thoracic erector spinae muscles, but these may be less accurate in patients who underwent lung surgery due to potential damage. Routine chest CT scans include images only up to the first lumbar level. Additionally, research on lung cancer patients suggested that the first lumbar erector spinae muscle provides a better prognosis than the pectoralis muscle.

We used the Hounsfield unit (HU) average value within the patient’s erector spinae muscle area on CT images to assess muscle mass, defining it as “HU_ESMcsa_”. First, to mitigate image quality variations due to patient size and scanning protocol, we denoised all chest CT images using commercial software. The HU_ESMcsa_ was then manually calculated by an experienced clinician and two researchers using in-house software that semi-automatically measures muscle and fat indices and calculates the HU range. Each measurement was performed twice per person to ensure accuracy and repeatability.

The process involved selecting the region of interest (ROI) within the erector spinae muscle area on CT images. Using in-house developed software, we manually calibrated the HU intensity range for muscle and adipose tissue. The HU dividing points were set to −30 at 120 kVp based on previous studies. Average intensity HU values were measured and validated against standard literature to account for variations in scanning protocols.

We applied a modified flood fill technique to precisely delineate the ROI, avoiding boundary edge areas that might include inhomogeneous intensity. This method allowed us to calculate the muscle and adipose tissue distribution, determining muscle density or “muscle index” by interpreting the mixture of muscle and fat within the selected ROI.

### Statistical analysis

Statistical analyses were performed using SPSS version 24.0 (SPSS Inc., Chicago, IL, United States). Continuous variables are presented as the mean ± standard deviation, while categorical variables are shown as counts and percentages. The Student’s *t*-test was employed for comparisons of continuous variables. Categorical variables were compared using the chi-square test or, when the expected number of events was fewer than five, the Fisher exact test. A *p*-value of less than 0.05 was considered statistically significant.

**Figure 2:**
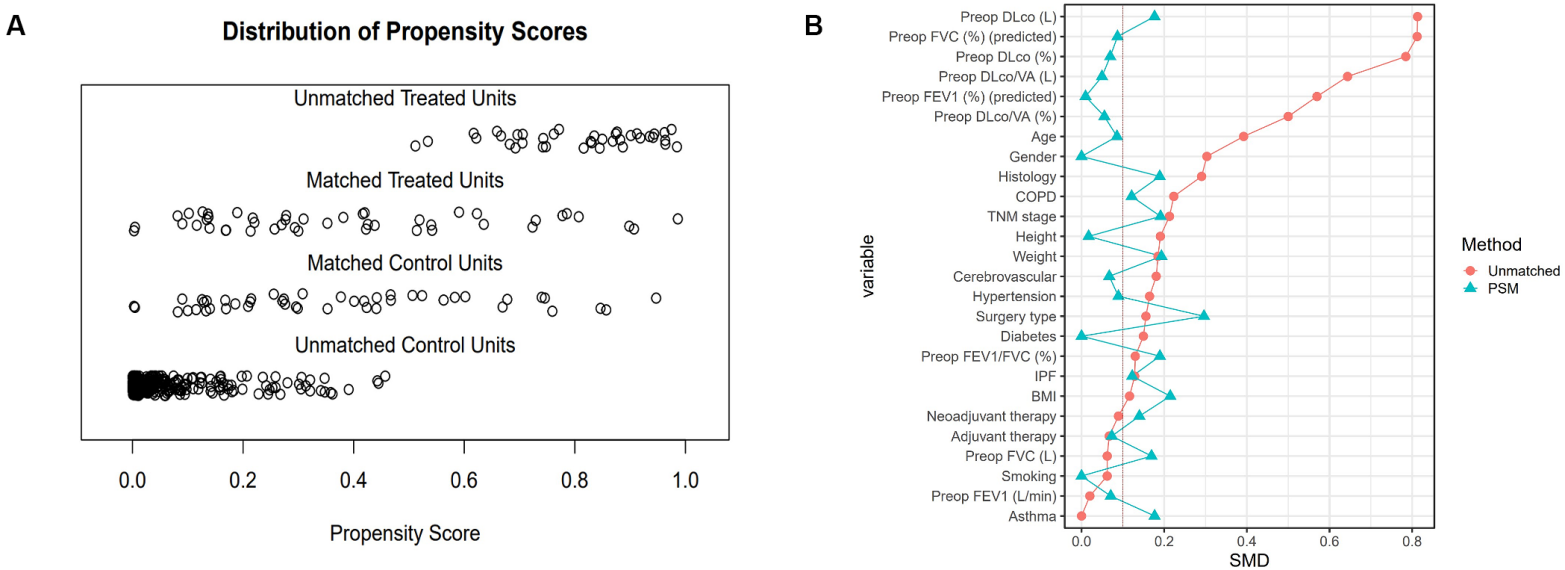
Distribution of propensity scores and standardized mean differences before and after PSM. **(A)** Propensity score distribution showing unmatched treated units, matched treated units, matched control units, and unmatched control units. After matching, the propensity scores of treated and control units align more closely, indicating improved balance. **(B)** Standardized mean differences (SMD) for each variable before and after PSM. Red dots represent the SMDs before matching, and blue triangles represent the SMDs after matching. The reduction in SMDs post-matching indicates successful balancing of the baseline characteristics between the PR and CTRL groups. PSM, propensity score matching; PR, pulmonary rehabilitation; CTRL, control; SMD, standardized mean difference; Preop, preoperative; FEV1, forced expiratory volume in 1 second; FVC, forced vital capacity; DLco, diffusing capacity of the lungs for carbon monoxide; VA, alveolar volume; COPD, chronic obstructive pulmonary disease; IPF, idiopathic pulmonary fibrosis; BMI, body mass index.

Pulmonary rehabilitation is generally recommended for patients with compromised lung function before undergoing lung surgery. This resulted in differences in baseline characteristics and pulmonary function between the two groups. To address these disparities, propensity score matching was used. Propensity scores were determined for each patient via multivariable logistic regression, considering covariates such as age, sex, height, weight, FEV1 (%), DLCO (%), comorbidities, cancer-related treatment (including neoadjuvant chemotherapy and neoadjuvant concurrent chemoradiotherapy), surgery type, and operation site. The nearest-neighbor method was utilized for 1:1 matching to ensure the most comparable propensity scores, and the effect size of the standardized mean difference (d) was calculated to evaluate the appropriateness of the propensity score matching (Figure 2).

## Results

### Baseline demographic and clinical characteristics

Before PSM, significant differences were observed between the PR and CTRL groups in several baseline characteristics. The mean age was higher in the PR group (57.29 vs. 51.92 years, *p* = 0.001, SMD = 0.392). The gender distribution also differed, with fewer females in the PR group (25.0% vs. 39.0%, *p* = 0.024, SMD = 0.303). There were no significant differences in height, weight, BMI, smoking status, COPD, asthma, IPF, hypertension, diabetes, cerebrovascular history, histological subtypes, TNM stages, neoadjuvant and adjuvant therapies, or surgery types between the groups before matching (Table 1).

**Table 1:** Comparison of baseline characteristics and demographics between PR and CTRL groups pre-and post-propensity score matching.

Variables	Level	Before PSM				After PSM			
		CTRL group (*n* = 336)	PR group (*n* = 84)	*p* value	SMD	CTRL group (*n* = 46)	PR group (*n* = 46)	*p* value	SMD
Age (y)		51.92 (13.43)	57.29 (13.94)	0.001	0.392	54.98 (14.23)	56.17 (13.65)	0.682	0.086
Gender (%)	Female	131 (39.0)	21 (25.0)	0.024	0.303	13 (28.3)	13 (28.3)	1	<0.001
	Male	205 (61.0)	63 (75.0)			33 (71.7)	33 (71.7)		
Height		170.07 (9.10)	171.75 (8.49)	0.126	0.191	172.28 (9.01)	172.13 (8.74)	0.935	0.017
Weight		70.62 (13.53)	73.06 (12.79)	0.137	0.185	75.22 (14.33)	72.52 (13.53)	0.356	0.193
BMI		24.50 (3.73)	24.94 (3.76)	0.341	0.116	25.46 (4.19)	24.61 (3.71)	0.306	0.215
Smoking (%)	Current smoker	27 (8.0)	8 (9.5)	0.875	0.062	4 (8.7)	4 (8.7)	1	<0.001
	Ex-smoker	119 (35.4)	28 (33.3)			14 (30.4)	14 (30.4)		
	Never-smoker	190 (56.5)	48 (57.1)			28 (60.9)	28 (60.9)		
COPD (%)	No	259 (77.1)	72 (85.7)	0.114	0.223	38 (82.6)	40 (87.0)	0.772	0.121
	Yes	77 (22.9)	12 (14.3)			8 (17.4)	6 (13.0)		
Asthma (%)	No	328 (97.6)	82 (97.6)	1	<0.001	42 (91.3)	44 (95.7)	0.673	0.177
	Yes	8 (2.4)	2 (2.4)			4 (8.7)	2 (4.3)		
IPF (%)	No	328 (97.6)	80 (95.2)	0.421	0.129	45 (97.8)	44 (95.7)	1	0.123
	Yes	8 (2.4)	4 (4.8)			1 (2.2)	2 (4.3)		
Hypertension (%)	No	215 (64.0)	47 (56.0)	0.217	0.165	29 (63.0)	27 (58.7)	0.831	0.089
	Yes	121 (36.0)	37 (44.0)			17 (37.0)	19 (41.3)		
Diabetes (%)	No	283 (84.2)	75 (89.3)	0.319	0.15	40 (87.0)	40 (87.0)	1	<0.001
	Yes	53 (15.8)	9 (10.7)			6 (13.0)	6 (13.0)		
Cerebrovascular (%)	No	304 (90.5)	71 (84.5)	0.167	0.181	41 (89.1)	40 (87.0)	1	0.067
	Yes	32 (9.5)	13 (15.5)			5 (10.9)	6 (13.0)		
Histology (%)	Adenocarcinoma	156 (46.4)	51 (60.7)	0.063	0.29	21 (45.7)	25 (54.3)	0.665	0.189
	Others	84 (25.0)	16 (19.0)			12 (26.1)	9 (19.6)		
	Squamous cell carcinoma	96 (28.6)	17 (20.2)			13 (28.3)	12 (26.1)		
TNM stage (%)	I	100 (29.8)	20 (23.8)	0.37	0.213	14 (30.4)	14 (30.4)	0.841	0.191
	II	140 (41.7)	32 (38.1)			17 (37.0)	17 (37.0)		
	III	83 (24.7)	27 (32.1)			11 (23.9)	13 (28.3)		
	IV	13 (3.9)	5 (6.0)			4 (8.7)	2 (4.3)		
Neoadjuvant therapy (%)	No	298 (88.7)	72 (85.7)	0.572	0.089	40 (87.0)	42 (91.3)	0.738	0.14
	Yes	38 (11.3)	12 (14.3)			6 (13.0)	4 (8.7)		
Adjuvant therapy (%)	No	303 (90.2)	74 (88.1)	0.717	0.067	42 (91.3)	41 (89.1)	1	0.073
	Yes	33 (9.8)	10 (11.9)			4 (8.7)	5 (10.9)		
Surgery type (%)	Lobectomy	132 (39.3)	32 (38.1)	0.645	0.156	20 (43.5)	20 (43.5)	0.578	0.296
	Pneumonectomy	9 (2.7)	2 (2.4)			1 (2.2)	0 (0.0)		
	Segmentectomy	86 (25.6)	27 (32.1)			14 (30.4)	11 (23.9)		
	Wedge resection	109 (32.4)	23 (27.4)			11 (23.9)	15 (32.6)		

PSM, propensity score matching; CTRL, control; PR, pulmonary rehabilitation; SMD, standardized mean difference; COPD, chronic obstructive pulmonary disease; IPF, idiopathic pulmonary fibrosis; TNM, tumor, node, metastasis; y, years; BMI, body mass index.

**Figure 3:**
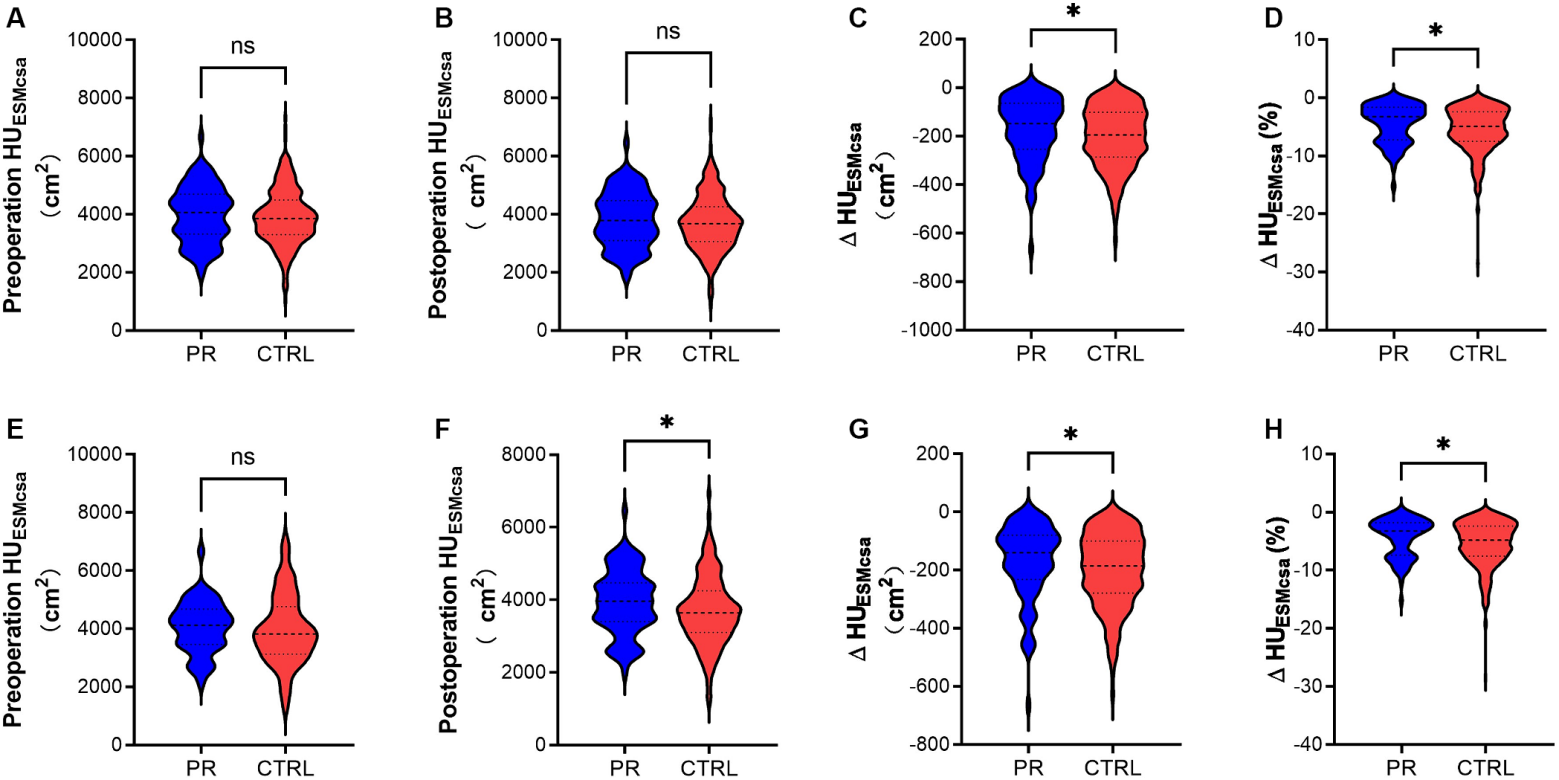
Changes in HUESMCSA of the PR and CTRL groups before **(A–D)** and after PSM (E-H). **(A,E)** Show preoperative HUESMCSA (cm^2^) in the PR and CTRL groups with no significant difference (ns). **(B,F)** Display postoperative HUESMCSA (cm^2^), with a significant difference in **(F)** (*). **(C,G)** Illustrate the change in HUESMCSA (ΔHUESMCSA) (cm^2^) between preoperative and postoperative measurements, showing significant differences in both (*). **(D,H)** Depict the percentage change in HUESMCSA (ΔHUESMCSA %) between preoperative and postoperative measurements, also indicating significant differences (*). * Indicates *p* < 0.05. ns indicates no significant difference. Data are presented as violin plots, with each plot displaying the distribution, median, and quartiles of the data. HUESMCSA, Hounsfield unit erector spinae muscle cross-sectional area; PR, pulmonary rehabilitation; CTRL, control; PSM, propensity score matching.

After PSM, the baseline characteristics between the PR and CTRL groups were well-balanced with no significant differences in age (56.17 vs. 54.98 years, *p* = 0.682, SMD = 0.086) and identical gender distribution (28.3% female in both groups). Other variables such as height, weight, BMI, smoking status, COPD, asthma, IPF, hypertension, diabetes, cerebrovascular history, histological subtypes, TNM stages, neoadjuvant and adjuvant therapies, and surgery types also showed no significant differences post-matching, indicating successful balancing between the groups (Figure 3).

### Preoperative pulmonary function test results

Before PSM, significant differences were observed between the PR and CTRL groups in several preoperative pulmonary function test variables. The PR group had a lower predicted FEV1 percentage (79.51% vs. 89.49%, *p* < 0.001, SMD = 0.57) and a lower predicted FVC percentage (84.26% vs. 96.23%, *p* < 0.001, SMD = 0.812). The diffusing capacity of the lungs for carbon monoxide (DLco) was also significantly lower in the PR group both in absolute terms (14.24 L vs. 17.54 L, *p* < 0.001, SMD = 0.813) and as a percentage of predicted values (75.10% vs. 86.16%, *p* < 0.001, SMD = 0.785). Similarly, DLco per alveolar volume (DLco/VA) was lower in the PR group in both absolute values (3.21 L vs. 3.77 L, *p* < 0.001, SMD = 0.644) and percentage of predicted values (82.85% vs. 89.94%, *p* < 0.001, SMD = 0.5). The relatively lower FEV1 and FVC in the PR group can be attributed to the fact that we administered PR to patients with poorer respiratory conditions, aiming to improve their preoperative status. Despite these initial differences, the PSM provided well-balanced cohorts for subsequent analysis (Table 2).

**Table 2:** Preoperative pulmonary function test results for PR and CTRL groups before and after propensity score matching.

Variables	Before PSM				After PSM			
	CTRL group (*n* = 336)	PR group (*n* = 84)	*p* value	SMD	CTRL group (*n* = 46)	PR group (*n* = 46)	*p* value	SMD
FEV1/FVC (%)	64.38 (9.53)	63.11 (10.07)	0.279	0.13	64.53 (9.26)	62.65 (10.53)	0.366	0.19
FEV1 (L/min)	2.21 (0.62)	2.22 (0.63)	0.869	0.02	2.25 (0.67)	2.30 (0.62)	0.735	0.071
FEV1 (%) (predicted)	89.49 (20.04)	79.51 (14.56)	<0.001	0.57	81.65 (20.19)	81.82 (13.24)	0.963	0.01
FVC (L)	3.51 (1.15)	3.58 (1.14)	0.612	0.062	3.55 (1.15)	3.75 (1.16)	0.419	0.169
FVC (%) (predicted)	96.23 (15.00)	84.26 (14.49)	<0.001	0.812	87.22 (12.70)	88.32 (12.70)	0.678	0.087
DLco (L)	17.54 (4.85)	14.24 (3.05)	<0.001	0.813	15.19 (4.39)	14.52 (3.07)	0.399	0.177
DLco (%)	86.16 (14.42)	75.10 (13.77)	<0.001	0.785	78.05 (13.78)	78.94 (11.70)	0.74	0.069
DLco/VA (L)	3.77 (0.92)	3.21 (0.81)	<0.001	0.644	3.53 (0.87)	3.49 (0.60)	0.813	0.049
DLco/VA (%)	89.94 (14.70)	82.85 (13.62)	<0.001	0.5	83.97 (15.97)	83.15 (13.22)	0.791	0.055

PSM, propensity score matching; CTRL, control; PR, pulmonary rehabilitation; SMD, standardized mean difference; Preop, preoperative; FEV1, forced expiratory volume in 1 second; FVC, forced vital capacity; DLco, diffusing capacity of the lungs for carbon monoxide; VA, alveolar volume.

After PSM, the PR and CTRL groups were well-balanced with no significant differences in any preoperative pulmonary function test variables. FEV1/FVC percentage (62.65% vs. 64.53%, *p* = 0.366, SMD = 0.19), absolute FEV1 (2.30 L vs. 2.25 L, *p* = 0.735, SMD = 0.071), and predicted FEV1 percentage (81.82% vs. 81.65%, *p* = 0.963, SMD = 0.01) were similar between groups. Likewise, FVC (3.75 L vs. 3.55 L, *p* = 0.419, SMD = 0.169) and predicted FVC percentage (88.32% vs. 87.22%, *p* = 0.678, SMD = 0.087) showed no significant differences. DLco values, both absolute (14.52 L vs. 15.19 L, *p* = 0.399, SMD = 0.177) and predicted percentage (78.94% vs. 78.05%, *p* = 0.74, SMD = 0.069), as well as DLco/VA values in absolute terms (3.49 L vs. 3.53 L, *p* = 0.813, SMD = 0.049) and predicted percentage (83.15% vs. 83.97%, *p* = 0.791, SMD = 0.055), were balanced post-matching.

### Exercise capacity, peak exercise symptoms, and HRQL

Before PSM, the PR group demonstrated significantly higher maximal WR in watts (86.24 vs. 78.01, *p* = 0.01, SMD = 0.331) and as a percentage (105.33% vs. 92.88%, *p* = 0.001, SMD = 0.421). Similarly, peak oxygen uptake (VO2) in mL/min was significantly higher in the PR group (1180.96 vs. 1014.37, *p* < 0.001, SMD = 0.622) and as a percentage (83.71% vs. 78.61%, *p* = 0.011, SMD = 0.313). The PR group also reported less leg soreness during exercise (3.42 vs. 4.13, *p* < 0.001, SMD = 0.474) and a lower total CAT score (9.80 vs. 12.40, *p* < 0.001, SMD = 0.574). There were no significant differences in dyspnea during exercise (*p* = 0.115), cough, phlegm, chest tightness, limited activities, confidence in leaving home, sleeplessness, and lack of energy (Table 3).

**Table 3:** Exercise capacity, peak exercise symptoms, and HRQL in PR and CTRL groups before and after propensity score matching.

Variables	Before PSM				After PSM			
	CTRL group (*n* = 336)	PR group (*n* = 84)	*p* value	SMD	CTRL group (*n* = 46)	PR group (*n* = 46)	*p* value	SMD
Maximal WR (watt)	78.01 (26.60)	86.24 (22.98)	0.01	0.331	73.33 (30.12)	81.67 (19.58)	0.119	0.328
Maximal WR (%)	92.88 (28.84)	105.33 (30.35)	0.001	0.421	90.00 (29.94)	104.76 (28.23)	0.017	0.507
Peak VO2 (mL/min)	1014.37 (255.51)	1180.96 (279.94)	<0.001	0.622	1004.74 (252.51)	1150.70 (269.62)	0.009	0.559
Peak VO2 (%)	78.61 (16.54)	83.71 (16.07)	0.011	0.313	78.04 (18.24)	82.46 (15.86)	0.219	0.258
Leg soreness during exercise	4.13 (1.47)	3.42 (1.49)	<0.001	0.474	4.15 (1.43)	3.33 (1.46)	0.007	0.572
Dyspnea during exercise	4.50 (1.75)	4.16 (2.01)	0.115	0.184	4.14 (1.90)	4.28 (1.97)	0.727	0.073
Total CAT score	12.40 (5.38)	9.80 (3.48)	<0.001	0.574	13.18 (5.55)	10.17 (3.96)	0.003	0.627
Cough	2.01 (0.10)	2.01 (0.11)	0.807	0.029	2.00 (0.10)	2.02 (0.10)	0.349	0.196
Phlegm	1.40 (0.10)	1.40 (0.09)	0.921	0.013	1.40 (0.12)	1.40 (0.08)	1	<0.001
Chest tightness	1.80 (0.10)	1.80 (0.10)	0.553	0.073	1.78 (0.09)	1.81 (0.09)	0.226	0.254
Breathlessness	1.90 (0.10)	1.51 (0.11)	<0.001	3.599	1.90 (0.11)	1.52 (0.10)	<0.001	3.78
Limited activities	0.94 (0.62)	0.79 (0.63)	0.05	0.238	0.90 (0.52)	0.77 (0.60)	0.269	0.232
Confidence in leaving home	0.89 (0.53)	0.69 (0.38)	0.001	0.43	0.91 (0.61)	0.71 (0.36)	0.054	0.407
Sleeplessness	1.66 (0.91)	1.31 (0.83)	0.001	0.401	1.70 (0.75)	1.34 (0.83)	0.032	0.455
Lack of energy	1.41 (0.92)	1.25 (0.83)	0.147	0.182	1.42 (0.91)	1.20 (0.87)	0.245	0.244

PSM, propensity score matching; CTRL, control; PR, pulmonary rehabilitation; SMD, standardized mean difference; WR, work rate; VO2, oxygen uptake; CAT, chronic obstructive pulmonary disease assessment test.

After PSM, the PR group continued to show significant improvements in several parameters. The maximal WR percentage remained higher (104.76% vs. 90.00%, *p* = 0.017, SMD = 0.507), and peak VO2 in mL/min (1150.70 vs. 1004.74, *p* = 0.009, SMD = 0.559) was significantly better in the PR group. Leg soreness during exercise was significantly lower (3.33 vs. 4.15, *p* = 0.007, SMD = 0.572), and the total CAT score was reduced (10.17 vs. 13.18, *p* = 0.003, SMD = 0.627). Breathlessness showed a marked improvement in the PR group both before (*p* < 0.001) and after PSM (*p* < 0.001). Other variables such as dyspnea during exercise, cough, phlegm, chest tightness, limited activities, confidence in leaving home, sleeplessness, and lack of energy did not show significant differences between the groups post-matching, indicating a good balance in these aspects.

### Postoperative pulmonary function test results

Before PSM, the PR group exhibited significantly better postoperative pulmonary function compared to the CTRL group. This included higher FEV1/FVC percentage (63.27% vs. 50.96%, *p* < 0.001, SMD = 0.904), FEV1 in liters per minute (2.25 vs. 1.74, *p* < 0.001, SMD = 0.705), and predicted FEV1 percentage (81.02% vs. 70.68%, *p* < 0.001, SMD = 0.489). Similarly, the PR group had higher FVC in liters (3.75 vs. 2.80, *p* < 0.001, SMD = 0.762) and predicted FVC percentage (84.32% vs. 76.10%, *p* < 0.001, SMD = 0.393). While the DLco in absolute terms was not significantly different (*p* = 0.077), the predicted DLco percentage was higher in the PR group (75.02% vs. 68.44%, *p* = 0.001, SMD = 0.383). DLco/VA was lower in the PR group both in absolute values (2.53 vs. 2.97, *p* < 0.001, SMD = 0.545) and as a percentage of predicted values (63.60% vs. 71.22%, *p* < 0.001, SMD = 0.517) (Table 4).

**Table 4:** Postoperative pulmonary function test data at rest and during exercise for PR and CTRL groups before and after propensity score matching.

Variables	Before PSM				After PSM			
	CTRL group (*n* = 336)	PR group (*n* = 84)	*p* value	SMD	CTRL group (*n* = 46)	PR group (*n* = 46)	*p* value	SMD
FEV1/FVC (%)	50.96 (10.60)	63.27 (16.08)	<0.001	0.904	50.87 (10.32)	64.17 (18.01)	<0.001	0.906
FEV1 (L/min)	1.74 (0.57)	2.25 (0.86)	<0.001	0.705	1.75 (0.57)	2.31 (0.80)	<0.001	0.806
FEV1 (%) (postdicted)	70.68 (20.50)	81.02 (21.73)	<0.001	0.489	64.32 (18.52)	84.45 (21.73)	<0.001	0.997
FVC (L)	2.80 (1.07)	3.75 (1.40)	<0.001	0.762	2.79 (0.98)	3.92 (1.32)	<0.001	0.968
FVC (%) (postdicted)	76.10 (17.64)	84.32 (23.77)	<0.001	0.393	68.30 (15.02)	88.75 (23.55)	<0.001	1.036
DLco (L)	13.87 (4.56)	14.85 (4.53)	0.077	0.217	11.90 (3.69)	15.50 (4.59)	<0.001	0.863
DLco (%)	68.44 (15.33)	75.02 (18.82)	0.001	0.383	60.46 (14.44)	78.66 (16.47)	<0.001	1.175
DLco/VA (L)	2.97 (0.86)	2.53 (0.74)	<0.001	0.545	2.72 (0.80)	2.75 (0.59)	0.871	0.034
DLco/VA (%)	71.22 (16.17)	63.60 (13.17)	<0.001	0.517	65.53 (15.59)	64.33 (12.94)	0.69	0.083
MIP (cmH20)	74.57 (25.60)	78.01 (24.52)	0.268	0.137	76.41 (24.77)	77.53 (25.61)	0.831	0.045
MEP (cmH2O)	112.26 (31.58)	110.39 (30.48)	0.625	0.06	106.72 (35.49)	108.82 (29.56)	0.758	0.064
VT (mL) (at rest)	501.88 (145.42)	501.19 (142.23)	0.969	0.005	459.35 (137.48)	529.57 (133.43)	0.015	0.518
VT (mL) (during exercise)	1108.84 (315.15)	1245.95 (304.78)	<0.001	0.442	1190.00 (286.21)	1260.65 (308.08)	0.258	0.238
RF (breaths/min) (at rest)	19.66 (4.92)	20.10 (5.06)	0.475	0.087	19.74 (5.20)	20.15 (5.55)	0.714	0.077
RF (breaths/min) (during exercise)	34.60 (6.10)	35.73 (5.58)	0.126	0.192	33.83 (6.07)	35.33 (5.85)	0.231	0.252
SpO2 (%) (at rest)	94.09 (2.63)	93.56 (2.72)	0.102	0.198	93.91 (2.86)	93.76 (2.68)	0.793	0.055
SpO2 (%) (during exercise)	93.08 (2.69)	92.50 (2.82)	0.081	0.211	92.89 (2.73)	92.65 (2.85)	0.682	0.086

PSM, propensity score matching; CTRL, control; PR, pulmonary rehabilitation; SMD, standardized mean difference; FEV1, forced expiratory volume in 1 second; FVC, forced vital capacity; DLco, diffusing capacity of the lungs for carbon monoxide; VA, alveolar volume; MIP, maximum inspiratory pressure; MEP, maximum expiratory pressure; VT, tidal volume; RF, respiratory frequency.

After PSM, the PR group continued to show significantly better postoperative pulmonary function results. The differences in FEV1/FVC percentage (64.17% vs. 50.87%, *p* < 0.001, SMD = 0.906), FEV1 in liters per minute (2.31 vs. 1.75, *p* < 0.001, SMD = 0.806), predicted FEV1 percentage (84.45% vs. 64.32%, *p* < 0.001, SMD = 0.997), FVC in liters (3.92 vs. 2.79, *p* < 0.001, SMD = 0.968), and predicted FVC percentage (88.75% vs. 68.30%, *p* < 0.001, SMD = 1.036) remained statistically significant. Additionally, DLco in both absolute terms (15.50 vs. 11.90, *p* < 0.001, SMD = 0.863) and predicted percentage (78.66% vs. 60.46%, *p* < 0.001, SMD = 1.175) showed significant improvement in the PR group. No significant differences were found in DLco/VA values post-matching. There were no significant differences in MIP, MEP, resting VT, RF, or SpO2 at rest or during exercise between the groups post-matching, indicating balanced postoperative characteristics.

### Postoperative cardiovascular responses to exercise

Before PSM, there were significant differences between the PR and CTRL groups in certain cardiovascular responses to exercise. The PR group had a lower CI during exercise (6.41 vs. 7.71 L/min/m^2^, *p* < 0.001, SMD = 0.681) and higher oxygen pulse (O2P) (9.23 vs. 8.48 mL/beat, *p* = 0.001, SMD = 0.393). SVI at rest and during exercise, CI at rest, WE, AT, HR and mean BP during exercise showed no significant differences before matching (Table 5).

**Table 5:** Postoperative cardiovascular responses to exercise in PR and CTRL groups.

Variables	Before PSM				After PSM			
	CTRL group (*n* = 336)	PR group (*n* = 84)	*p* value	SMD	CTRL group (*n* = 46)	PR group (*n* = 46)	*p* value	SMD
SVI (ml/min/m^2^) (at rest)	41.98 (10.16)	42.67 (9.74)	0.575	0.069	44.51 (10.30)	42.42 (9.34)	0.311	0.212
SVI (ml/min/m^2^) (during exercise)	54.40 (14.34)	50.83 (17.57)	0.052	0.223	55.42 (13.52)	51.71 (17.07)	0.25	0.241
CI (L/min/m^2^) (at rest)	3.44 (0.67)	3.39 (0.58)	0.478	0.091	3.46 (0.62)	3.30 (0.58)	0.215	0.26
CI (L/min/m^2^) (during exercise)	7.71 (1.91)	6.41 (1.92)	<0.001	0.681	7.87 (1.83)	6.24 (1.90)	<0.001	0.869
O2P (mL/beat)	8.48 (1.86)	9.23 (1.94)	0.001	0.393	8.51 (1.52)	8.99 (1.87)	0.176	0.285
WE (mL/min/W)	9.02 (1.93)	9.15 (2.06)	0.569	0.068	8.58 (1.87)	8.77 (2.03)	0.643	0.097
AT (mL/min)	679.64 (138.62)	705.83 (155.61)	0.132	0.178	669.78 (150.13)	699.78 (155.84)	0.35	0.196
HR (beats/min) (during exercise)	131.19 (20.88)	127.69 (16.86)	0.155	0.185	132.26 (19.38)	125.98 (14.81)	0.084	0.364
Mean BP (mmHg) (during exercise)	108.99 (14.78)	107.12 (13.85)	0.294	0.131	112.22 (15.06)	108.63 (14.72)	0.251	0.241

PSM, propensity score matching; CTRL, control; PR, pulmonary rehabilitation; SMD, standardized mean difference; SVI, stroke volume index; CI, cardiac index; O2P, oxygen pulse; WE, work efficiency; AT, anaerobic threshold; HR, heart rate; BP, blood pressure.

After PSM, the PR group continued to show a significantly lower CI during exercise (6.24 vs. 7.87 L/min/m^2^, *p* < 0.001, SMD = 0.869). No significant differences were observed in other variables, including SVI at rest (*p* = 0.311) and during exercise (*p* = 0.25), CI at rest (*p* = 0.215), O2P (*p* = 0.176), WE (*p* = 0.643), AT (*p* = 0.35), HR during exercise (*p* = 0.084), and mean BP during exercise (*p* = 0.251). This indicates a good balance between the PR and CTRL groups in terms of postoperative cardiovascular responses after matching.

### Muscle measurements and changes post-surgery

In our analysis of muscle measurements, we found no significant differences between the PR and CTRL groups in preoperative and postoperative muscle cross-sectional area (CSA) in HU_ESMCSA_. However, when examining the changes post-surgery, the PR group experienced significantly smaller reductions in both absolute HU_ESMCSA_ values and percentage changes compared to the CTRL group. Specifically, the PR group showed less decline in ΔHU_ESMCSA_ and percentage change in two different muscle groups, indicating that pulmonary rehabilitation was effective in mitigating muscle loss post-surgery. These findings highlight the potential benefits of PR in preserving muscle mass in lung surgery patients.

## Discussion

Our study demonstrated significant improvements in exercise capacity, HRQL, and cardiopulmonary function in patients with NSCLC who underwent pulmonary rehabilitation. Notably, the PR group showed higher values in FEV1/FVC, FEV1, predicted FVC percentage, and maximal WR percentage post-surgery. Furthermore, patients in the PR group exhibited better cardiovascular responses, including a lower CI during exercise and higher peak oxygen uptake (VO2). Muscle measurements indicated significantly smaller reductions in ΔHU_ESMCSA_, highlighting the role of PR in mitigating postoperative muscle loss. These findings underline the effectiveness of PR in enhancing postoperative recovery and overall physical function in lung cancer patients. The relationship between PR and the ERAS protocol is complementary, with each addressing different aspects of patient care. PR focuses on respiratory and physical rehabilitation, improving muscle strength, exercise capacity, and quality of life. ERAS encompasses broader perioperative care, including pain management, nutritional support, and early mobilization, to enhance recovery and reduce hospital stay. Together, they offer a comprehensive strategy to optimize patient outcomes and reduce complications, highlighting the importance of incorporating both protocols in managing lung surgery patients (13).

The improvements in exercise capacity and cardiopulmonary function observed in our study align with previous research highlighting the benefits of PR in patients with chronic respiratory diseases (14–16). Huang et al. reported that PR significantly improved exercise capacity, HRQL, and cardiopulmonary function in lung cancer patients, with increased peak VO2 and WR, reduced exertional symptoms, and enhanced respiratory muscle strength (17). Our findings are consistent with these results, indicating that PR can lead to substantial enhancements in physical performance and quality of life for lung cancer patients undergoing surgery. Pulmonary function parameters, such as FEV1/FVC ratio and FVC, showed significant improvement in the PR group, corroborating the results of previous studies on the positive effects of PR on lung function. Wang et al. (10) conducted a meta-analysis that demonstrated PR’s efficacy in improving postoperative clinical status in patients with lung cancer and COPD, showing enhanced pulmonary function and reduced postoperative complications. PR is known to reduce the risk of postoperative complications, although this aspect was not initially discussed in our paper. PR improves respiratory muscle strength, enhances exercise capacity, and promotes overall recovery, which can collectively reduce the incidence of postoperative complications. Similarly, our study revealed significant improvements in FEV1 and FVC percentages, suggesting that PR can effectively enhance lung function and aid in postoperative recovery.

The cardiovascular benefits of PR observed in our study, including improved CI and O2P during exercise, further emphasize the comprehensive impact of PR on patients’ overall health. Exercise training, a core component of PR, has been shown to improve cardiac function and enhance oxygen delivery to tissues, contributing to better exercise performance and reduced symptoms (18–20). Our findings align with this evidence, demonstrating that PR not only benefits respiratory function but also significantly improves cardiovascular performance, which is crucial for enhancing overall physical capacity and quality of life in lung cancer patients (21–23).

Our study also highlighted the importance of PR in mitigating muscle loss post-surgery. The PR group experienced significantly smaller reductions in ΔHU_ESMCSA_ compared to the CTRL group, indicating that PR helps preserve muscle mass during the postoperative period. This finding is consistent with research by Illini et al. (24), who reported that PR effectively preserves muscle mass and improves physical function in lung cancer patients following surgery. The preservation of muscle mass is crucial for maintaining physical strength and function, reducing the risk of complications, and enhancing the overall recovery process (25–27).

Despite the significant findings, our study has several limitations. First, the sample size is relatively small, and the single-center design may introduce selection bias. Future multicenter studies with larger sample sizes are necessary to validate our findings. Second, Our study included patients with relatively better baseline pulmonary function compared to typical rehabilitation cohorts. This selection bias is inherent due to the retrospective nature of our study and the inclusion criteria we applied. Specifically, patients were chosen based on their ability to participate in the rehabilitation program and to minimize the impact of severe comorbidities, which resulted in higher baseline FEV1 and DLCO values. This criterion ensured that the participants could safely engage in the intensive exercise components of the pulmonary rehabilitation program. Consequently, the outcomes observed in this study may not fully represent the broader population of lung surgery patients, particularly those with more compromised pulmonary function. This limitation highlights the need for further prospective studies to evaluate the effects of pulmonary rehabilitation in a more diverse and representative patient population, including those with more severe baseline pulmonary impairments. Additionally, the relatively short follow-up period of 12 weeks may not be sufficient to determine the long-term effects of PR. Longer follow-up studies are required to confirm the sustained benefits of PR. Finally, all patients in this study had NSCLC, and the results may not be generalizable to patients with small cell lung cancer (SCLC), who may have different treatment responses and prognoses. Another limitation of our study is the type of surgical procedure performed. Specifically, 32% of the patients underwent a wedge resection, a non-anatomical resection with debated oncological benefits. This decision was based on clinical judgment regarding the tumor’s location, size, and the patient’s health status. While necessary for some patients, this introduces variability that may affect the generalizability of our findings.

## Conclusion

In summary, our study underscores the significant benefits of PR in improving exercise capacity, HRQL, and cardiopulmonary function in lung cancer patients. PR also plays a vital role in preserving muscle mass post-surgery, contributing to better physical function and recovery. These findings highlight the importance of incorporating PR into the standard care of lung cancer patients undergoing surgery to enhance their overall health outcomes and quality of life.

## Data availability statement

The raw data supporting the conclusions of this article will be made available by the authors, without undue reservation.

## Ethics statement

The studies involving humans were approved by the Ethics Committee of Shanghai Second Rehabilitation Hospital. The studies were conducted in accordance with the local legislation and institutional requirements. The ethics committee/institutional review board waived the requirement of written informed consent for participation from the participants or the participants’ legal guardians/next of kin because Given the retrospective nature of the study, the requirement for informed consent was waived.

## Author contributions

CN: Conceptualization, Formal analysis, Investigation, Methodology, Visualization, Writing – original draft. HL: Formal analysis, Methodology, Validation, Writing – review & editing. ZZ: Data curation, Formal analysis, Methodology, Writing – review & editing. QW: Conceptualization, Data curation, Resources, Visualization, Writing – review & editing. YW: Project administration, Supervision, Validation, Writing – review & editing.

## Glossary

PR: Pulmonary Rehabilitation
HRQL: Health-Related Quality of Life
NSCLC: Non-Small Cell Lung Cancer
PSM: Propensity Score Matching
CTRL: Control
FEV1: Forced Expiratory Volume in 1 Second
FVC: Forced Vital Capacity
CI: Cardiac Index
WR: Work Rate
VO2: Oxygen Uptake
ΔHU_ESMCSA_: Change in Hounsfield Unit Erector Spinae Muscle Cross-Sectional Area
COPD: Chronic Obstructive Pulmonary Disease
ILD: Interstitial Lung Disease
ATS: American Thoracic Society
CAT: Chronic Obstructive Pulmonary Disease Assessment Test
CPET: Cardiopulmonary Exercise Testing
VCO2: Carbon Dioxide Output
VT: Tidal Volume
RF: Respiratory Frequency
BP: Blood Pressure
AT: Anaerobic Threshold
WE: Work Efficiency
O2P: Oxygen Pulse
VEQ: Ventilatory Equivalent
MIP: Maximum Inspiratory Pressure
MEP: Maximum Expiratory Pressure
SVI: Stroke Volume Index
ROI: Region of Interest
HU: Hounsfield Unit
DLco: Diffusing Capacity of the Lungs for Carbon Monoxide
VA: Alveolar Volume
IPF: Idiopathic Pulmonary Fibrosis
BMI: Body Mass Index
SD: Standard Deviation
SpO2: Arterial Oxygen Saturation
SMD: Standardized Mean Difference
Preop: Preoperative
ERAS: Enhanced Recovery After Surgery
